# A digital platform-based stratified intervention for preventing disability risk in community-dwelling older adults: study protocol for a randomized multicenter trial

**DOI:** 10.3389/fmed.2026.1890107

**Published:** 2026-07-30

**Authors:** Yuping Zhang, Yitong Mao, Zhiting Guo, Wen Gao, Jiayi Wang, Jingfen Jin

**Affiliations:** 1 Nursing Department, The Second Affiliated Hospital of Zhejiang University School of Medicine (SAHZU), Hangzhou, Zhejiang, China; 2 School of Public Health and Nursing, Hangzhou Normal University, Hangzhou, Zhejiang, China

**Keywords:** digital intervention, disability risk, older adults, protocol, randomized control study

## Abstract

**Background:**

Population aging has sharply increased the prevalence of disability, imposing a substantial public health burden. Early identification of high-risk older adults and preventive interventions are crucial for promoting healthy ageing.

**Objective:**

This study aims to evaluate the effectiveness of a digital platform-based stratified intervention in delaying functional decline and reducing the incidence of disability among older adults in a real-world community setting.

**Methods:**

This multi-center, assessor-blind, randomized controlled trial will enroll 238 participants aged 65 years and older from eight healthcare community centers. Participants will be stratified by disability-risk level and randomized 1:1 to either a digital platform based, multi-domain intervention or usual care. The platform integrates evidence-based components, with intensity matched to low, moderate, or high risk of disability. Intervention lasts 6 months, with periodic reassessment of disability risk, with risk-dependent adjustment of the intervention plan. The primary outcome is the Short Physical Performance Battery (SPPB) score, while secondary outcomes include the incidence of disability, changes in disability risk scores, and adherence to the intervention.

**Conclusion:**

This study will evaluate the digital, risk-stratified, multidomain intervention designed to prevent disability in community-dwelling older adults. If effective, the findings will provide actionable evidence for integrating digital tools and personalized strategies into community-based geriatric care, advancing sustainable approaches to healthy aging and reducing the disability burden in rapidly aging societies.

**Study protocol registration:**

http://clinicaltrials.gov, Identifier NCT07508813.

## 1 Introduction

Due to the acceleration of aging and changes in disease spectrum, disability has become an increasingly severe public health problem (1). Functional disability is generally defined as dependency in performing daily activities which are categorized into basic activities of daily living (BADL) and instrumental activities of daily living (IADL) due to physiological, psychological, or social factors (2, 3). A growing number of older adults, as their ages advance, most typically experience a decline in health or a gradual loss of the ability to perform basic yet essential daily activities, such as bathing or doing housework (4). The prevalence of disability among older adults in China was 26.4%, which is higher than in the US (15.6%) and the UK (18.3%) (5, 6). Disability among older adults is associated with a substantial decline in quality of life, increased healthcare expenditures, elevated mortality, and considerable caregiving and economic burdens for families and society (6–9).

Early identification of individuals at risk of disability could help guide targeted intervention strategies (10), and the implementation of preventive interventions is essential for promoting healthy ageing. Generally, disability risk refers to the predicted likelihood of future functional decline or development of BADL and/or IADL limitations among older adults who have not yet developed established disability. While various tests and prediction algorithms have been used to classify older adults into different categories of functional risk (10, 11), in this study, disability risk is operationally assessed using a self-developed and validated risk prediction model based on multidimensional risk factors.

Traditionally, multidomain interventions—including physical exercise (12, 13), nutritional guidance (14), and chronic disease management (15)—have been proven effective in delaying functional decline among high-risk older adults. However, traditional community-based programs often face significant challenges, such as high human resource costs, limited geographical accessibility, and difficulty in maintaining long-term adherence. In the era of global ageing, digital technology has emerged as an important and useful tool to overcome various challenges associated with aging, such as reduced physical and cognitive function (16, 17). For instance, technology-supported programs such as the LETHE trial combine smartphone applications, smartwatches, and digital coaching to deliver multimodal lifestyle interventions targeting dementia risk reduction (18). Similarly, the INSPIRE ICOPE-CARE program in France implemented a large-scale digital screening and monitoring system based on the WHO Integrated Care for Older People (ICOPE) framework (19). These findings highlight the potential of digital health interventions to overcome traditional barriers (20). Nevertheless, the implementation of digital interventions in geriatric populations requires careful consideration of technological literacy and accessibility (21, 22). Older adults with lower educational attainment, limited financial means, or reduced digital literacy may face barriers to using smartphones or internet-based services, potentially leading to their exclusion. To address this digital divide (23), digital platforms for older adults should be designed with simplified interfaces and integrated with human support from community staff or family caregivers.

Beyond the challenges of digital accessibility, important evidence gaps persist in current digital health research. Most available interventions have been developed around a single disease or clinical scenario, for example, post-hip fracture rehabilitation or dementia prevention. Others focus on only selected aspects of physical or cognitive function. These approaches may overlook the comprehensive functional health status of older adults living in the community. Moreover, risk-based intervention pathways have rarely been embedded into digital platforms, making it difficult to deliver tailored support according to different levels of disability risk. Another limitation is that many previous studies were not conducted as rigorous multicenter randomized controlled trials, which weakens the external validity of their findings. More evidence is also needed on whether such interventions can be implemented in real-world community settings, especially in rapidly aging low- and middle-income countries such as China.

In response to these limitations, this study will develop a digital platform-based stratified intervention program for preventing disability risk in community-dwelling older adults. To address potential barriers related to technological literacy and accessibility, the intervention is designed as a supported model, where community health staff and family caregivers can assist older adults in engaging with the digital platform. Through a multicenter randomized trial, we aim to evaluate a digital health platform that integrates repeated risk assessment, predictive modeling, and individualized intervention recommendations. The specific aims are as follows: (1) to develop a stratified disability risk intervention program; (2) to determine whether stratified digital intervention can delay functional decline and reduce new-onset disability; and (3) to assess participants’ adherence to the digital platform under real-world community conditions.

## 2 Methods and analysis

### 2.1 Study design and settings

#### 2.1.1 Design

**Figure 1:**
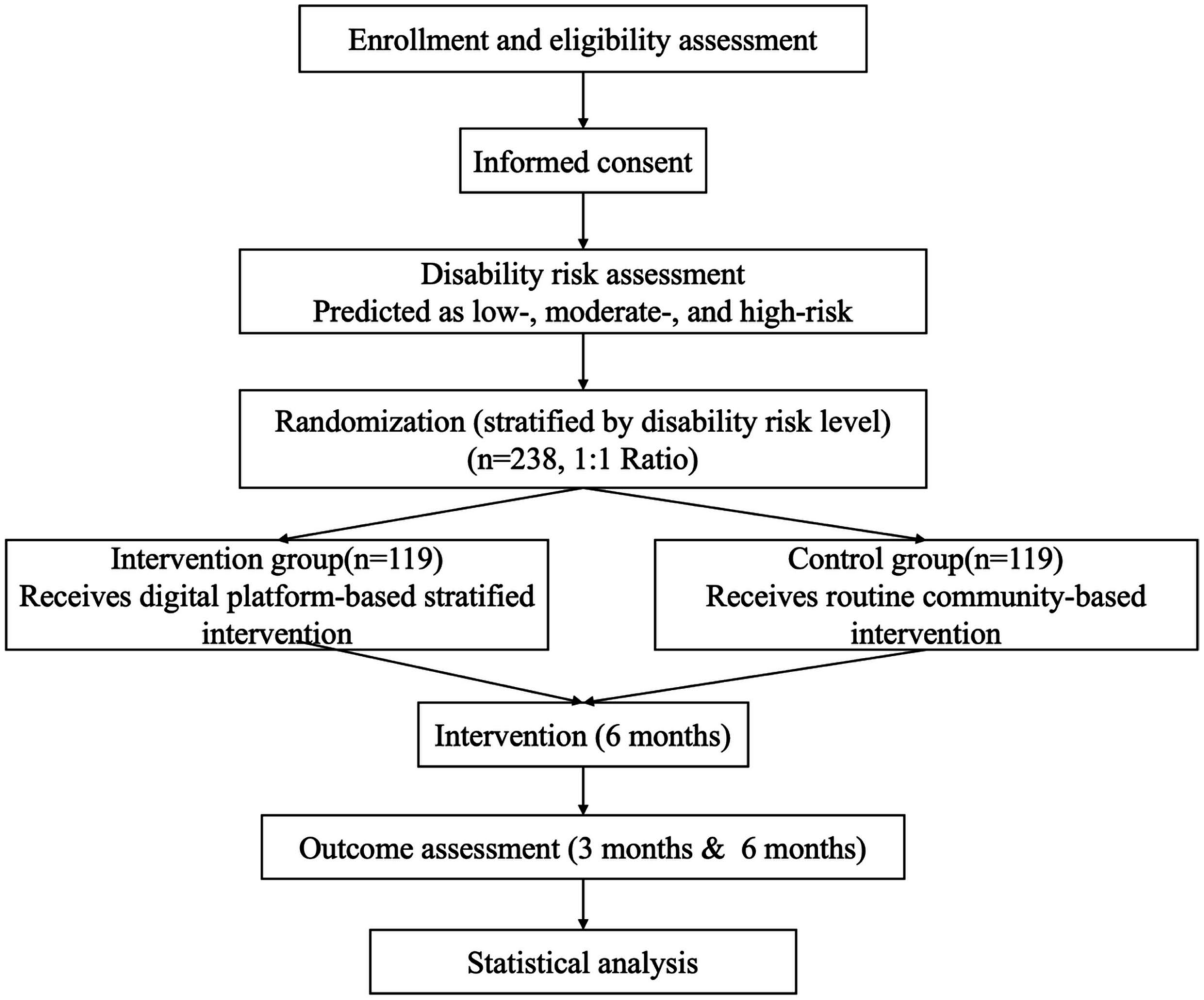
Study flowchart.

This study is designed as a randomized, assessor-blinded, parallel-group, multicenter trial. Eligible participants will be randomly allocated in a 1:1 ratio to either the intervention group or control group. The elderly in the intervention group will receive a digital platform-based stratified intervention, and the control group will receive usual care. To ensure a balanced distribution of baseline characteristics and minimize potential confounding effects, stratified randomization will be employed based on the level of disability risk. The study protocol is developed in accordance with the Standard Protocol Items: Recommendations for Interventional Trials (SPIRIT) guidelines (24) to ensure transparency, completeness, and rigor methodology. The study flowchart is depicted in Figure 1, and the trial schedule is outlined in Table 1. The trial has been registered at ClinicalTrials.gov (NCT07508813) on Sep 24, 2025.

**Table 1:** Time schedule of the trial process.

	Study period			
	Enrolment	Allocation	Post-allocation	
Time points	-t_1_	t_0_	t_1_	t_2_
Enrollment				
Eligibility screening	P			
Informed consent	P			
Disability risk factors collection	P			
Disability risk calculation	P			
Randomization and allocation		P		
Intervention			0–6 months	
Outcome measurement				
Short Physical Performance Battery (SPPB)		P	P	P
Incidence of disability				P
Disability risk score	P		P	P
Intervention adherence			P	P

#### 2.1.2 Settings

This trial will be implemented in eight community healthcare centers in Zhejiang Province, China. Of these, five are located in Hangzhou City, representing urban communities, and three are located in Changxing County, representing rural communities. The inclusion of both urban and rural sites is intended to improve the representativeness of the study population and strengthen the generalizability of the findings. All participating centers provide primary healthcare services and have established routine contact with older adults living in the community, which will support participant recruitment, delivery of the intervention, and follow-up assessments.

### 2.2 Participants

#### 2.2.1 Inclusion criteria

Participants meeting all the following criteria will be eligible for inclusion:

(1) Aged 65 years or older;
(2) Community-dwelling permanent residents;
(3) Able to use a smartphone independently or with assistance from a primary caregiver;
(4) Cognitively able to understand study procedures and communicate effectively;
(5) Willing to participate and able to provide written informed consent.

To ensure accurate enrollment, the study team will verify the eligibility criteria during the initial screening interview. Smartphone usage will be confirmed by asking the participant or their primary caregiver about smartphone ownership and their ability to perform basic application operations. Cognitive ability and communication skills will be assessed by trained researchers based on the participant’s ability to engage in the screening conversation and comprehend the study procedures during the informed consent process. Willingness to participate will be strictly documented through the signing of the written informed consent form.

#### 2.2.2 Exclusion criteria

Participants will be excluded if they meet any of the following criteria:

(1) Inability to independently perform Basic Activities of Daily Living (BADL) or Instrumental Activities of Daily Living (IADL) (25, 26). Any difficulty in performing one or more of these activities is considered indicative of disability.
(2) Significant cognitive impairment, defined as an MMSE score <10 (27) or a clinical diagnosis of dementia;
(3) Severe speech, hearing, or visual impairments that would interfere with communication or participation in the intervention;
(4) Severe or terminal illness limiting life expectancy.

A clinical diagnosis of dementia was verified through participant self-report, caregiver proxy report, or review of available medical history. Speech, hearing, or visual impairments that could interfere with communication or study participation were identified by study staff through observation and interaction during face-to-face screening. Severe or terminal illness was determined based on self-reported medical history or caregiver interview.

Although the enrolled participants are currently independent (free of BADL/IADL limitations), they remain at risk for future functional decline due to advanced age. By focusing on primary prevention, this study will utilize a validated prediction model to quantify the varying levels of underlying disability risk among this population.

#### 2.2.3 Termination and suspension criteria

Participants may be withdrawn from the study under the following conditions:

(1) Withdrawal of consent or refusal to continue participation during the study period;
(2) Loss to follow-up, defined as inability to contact the participant after three telephone attempts and failure to reach the participant through community healthcare workers;
(3) Occurrence of serious adverse events that necessitate discontinuation of participation.

#### 2.2.4 Recruitment strategy

To facilitate sufficient enrollment, the research team will recruit participants through multiple channels. Study information will be disseminated by displaying posters at community healthcare centers and activity centers. In addition, community health workers and family physicians will assist in identifying and contacting potentially eligible older adults. Health education activities will also be organized to introduce the study to the target population. Older adults who are willing to participate will subsequently be screened for eligibility by trained members of the research team. Written informed consent will be obtained from all participants prior to enrollment. Participants will be informed of the study objectives, procedures, potential risks, and their rights to withdraw at any time without penalty.

#### 2.2.5 Sample size calculation

The sample size calculation was based on the expected difference in Short Physical Performance Battery (SPPB) scores between the intervention and control groups. In a published study of community-dwelling older adults in China, the average baseline SPPB score of 1,014 older adults was (8.92 ± 2.11) (28). For the expected between-group difference, a 1-point difference in SPPB was selected as the target clinically meaningful effect based on previous evidence that most estimates of substantial change for the SPPB ranged from 0.99 to 1.34 points (29). On this basis, the mean SPPB score was assumed to be 8.92 in the control group and 9.92 in the intervention group. Using PASS software, and assuming a two-sided *α* of 0.05 together with a power of 90%, the required number of participants was estimated to be 95 per group, corresponding to 190 participants in total. Considering a potential dropout rate of 20%, the sample size was adjusted to 238 participants.

Preliminary data further suggested that the proportions of older adults at high, moderate, and low risk of disability were approximately 1:5:4. Based on this distribution, the final sample is expected to comprise about 24 high-risk participants, 120 moderate-risk participants, and 94 low-risk participants.

### 2.3 Randomization and blinding

#### 2.3.1 Randomization sequence

Before randomization, eligible participants will undergo a baseline disability risk assessment using a disability risk prediction model that we previously developed and externally validated. The model produces a continuous predicted probability of disability for each participant. Two cut-off values were identified using the maximally selected rank statistics method and applied to classify participants into low-, moderate-, and high-risk groups. Within each stratum, participants will be allocated in a 1:1 ratio to either the intervention group or the control group.

The stratified randomization sequences will be generated using SPSS software by an independent researcher who will not participate in recruitment, intervention implementation, or outcome evaluation. A separate randomization list will be prepared for each disability risk stratum to maintain balance between the intervention and control groups across different risk levels.

#### 2.3.2 Allocation concealment

Allocation concealment will be maintained through a centralized randomization process. The randomization lists will be kept securely by the independent researcher who will have no role in participant recruitment, enrollment, intervention delivery, or outcome evaluation, or data analysis.

After completion of the baseline assessment and confirmation of the participant’s disability risk level, the enrolling investigator will contact the independent researcher and provide the relevant risk-stratification information. The independent researcher will then assign the participant to the intervention or control group based on the corresponding pre-generated randomization list for that risk stratum. Members of the research team responsible for recruitment, baseline assessment, intervention delivery, and outcome evaluation will not have access to the randomization sequences before group assignment. This procedure is intended to preserve allocation concealment and reduce the risk of selection bias.

#### 2.3.3 Blinding

Given that the intervention is delivered through a digital platform, blinding of participants and intervention providers is impractical. The intervention component of the trial will therefore be conducted as an open-label procedure. However, blinding will be applied to outcome assessment and data analysis. Specifically, outcome assessors and statisticians will be unaware of participants’ group assignments until the primary analyses have been completed.

To reduce the possibility of unblinding, separate staff members will be assigned to intervention-related tasks and outcome evaluations. Outcome assessors will not be involved in intervention delivery, follow-up management, or access to the digital platform usage records. Group allocation information will not be included in outcome assessment forms or in the datasets provided to outcome assessors. Participants will be reminded at each assessment visit not to discuss or disclose their allocation status or intervention experience to the assessors. If accidental unblinding occurs, it will be documented, and another blinded assessor will complete the assessment or subsequent assessments where feasible. Data analysis will be performed using coded group labels until the primary analyses have been completed.

### 2.4 Interventions

#### 2.4.1 Intervention group

Participants in the intervention group will receive a digital platform-based stratified intervention program designed for disability risk prevention.

In this study, the Disability Risk Prevention Management Platform for Older Adults (DRPP) was developed by the Second Affiliated Hospital, Zhejiang University School of Medicine. It was constructed to provide community-dwelling older adults with integrated services for disability risk assessment, ongoing monitoring, and personalized intervention delivery.

The intervention model embedded in the platform was developed based on risk-stratified management and comprehensive geriatric care. Evidence-based recommendations were organized into four intervention domains, namely physical activity, nutrition, rest and sleep, and psychological and social support. Across these domains, 87 intervention components were identified and incorporated into the system as a structured knowledge base. These components were then converted into digital materials suitable for platform-based delivery.

The platform provides different intervention packages according to the participant’s level of disability risk. Among low-risk participants, the intervention focuses primarily on maintaining functional ability and promoting healthy behaviors, including regular exercise, adequate nutrition, good sleep practices, and engagement in social activities. For participants classified as moderate risk, the intervention places greater emphasis on structured exercise delivered at a higher frequency. Participants in the high-risk group receive a more intensive and continuous intervention package, with careful attention to safety throughout the process. In addition to disability risk level, intervention components were further adapted according to clinical and functional tailoring factors, including SPPB, frailty status, balance capacity, BMI, depression status, sleep and specific chronic conditions such as hypertension. The stratified intervention strategies are shown in Table 2. The measures of the tailoring factors are shown in Supplementary Table 1. The details of intervention contents are provided in Supplementary Table 2.

**Table 2:** Stratified intervention strategies for disability prevention in older adults.

Tailoring factors	Intervention component	Format (selectable based on individual preference)	Recommended dose / check-in frequency		
			High risk	Moderate risk	Low risk
/	Warm-up exercise	Marching in place / Joint mobility exercise / Dynamic stretching	10 min/session; accompanying exercise training		
SPPB ≥ 10	Aerobic exercise	Jogging / Stair climbing / Aerobic dance	20 min/session; 2 times/week	20 min/session; 2 times/week	20 min/session; 1 time/week
/		Brisk walking / Swimming / Baduanjin	30 min/session; 4 times/week	30 min/session; 3 times/week	30 min/session; 2 times/week
SPPB ≤ 9 or frailty		Walking / Housework / Tai Chi	50 min/session; 3 times/week	40 min/session; 3 times/week	40 min/session; 2 times/week
Frailty or upper limb muscle strength grade 3–4	Resistance exercise	Bodyweight exercises - chest expansion / straight arm abduction	10 reps/set, 3 sets/day; daily	10 reps/set, 2 sets/day; daily	10 reps/set, 2 sets/session; 3 times/week
		Bodyweight exercises - half-squat stand / heel raise / seated leg extension	10 reps/set, 3 sets/day; daily	10 reps/set, 2 sets/day; daily	10 reps/set, 2 sets/session; 3 times/week
Balance capacity grade ≤ 3; or history of falls / Parkinson’s disease / stroke	Balance exercise	Static balance exercise/ Dynamic balance exercise	15 min/session; 2 times/week	10 min/session; 2 times/week	10 min/session; 2 times/week
/	Cool-down exercises	Marching in place / Joint mobility exercise / Dynamic stretching	10 min/session; accompanying exercise training		
/	Dietary pattern^*^	Mediterranean diet	3 times/week	2 times/week	1 time/week
Frailty or muscle strength ≤ grade 3		High-protein diet	3 times/week	2 times/week	1 time/week
History of hypertension		DASH diet	3 times/week	2 times/week	1 time/week
BMI ≤ 18.5 (age <70) or BMI ≤ 20 (age ≥70)		High-calorie diet	3 times/week	2 times/week	1 time/week
History of diabetes		Low-glycemic diet	3 times/week	2 times/week	1 time/week
History of digestive disease		Age-friendly diet	3 times/week	2 times/week	1 time/week
Frailty or muscle strength ≤ grade 3	Nutrient supplement ^*^	Protein(1.2 g/kg/d)	3 times/week	2 times/week	1 time/week
Female: age ≥65; Male: age >70		Calcium(1,300 mg/d)	3 times/week	2 times/week	1 time/week
Male: 65 < age≤70		Calcium(1,000 mg/d)	3 times/week	2 times/week	1 time/week
Age ≥ 65		Vitamin D(800 U)	3 times/week	2 times/week	1 time/week
/	Sleep guidance	Healthy sleep education	1 time/week		
PSQI ≥ 7	Relaxation and meditation	Relaxation and meditation training	15 min/session; 1 time/week		
MMSE ≤ 20 (primary school or below) or MMSE ≤ 24 (middle school or above) or self-rated memory fair or poor	Memory training	Episodic memory / working memory / associative memory training	20 min/session; 3 times/week	15 min/session; 3 times/week	15 min/session; 2 times/week
MMSE ≤ 20 (primary school or below) or MMSE ≤ 24 (middle school or above)	Attention training	Dual-task walking/ deep breathing exercise	20 min/session; 3 times/week	10 min/session; 3 times/week	10 min/session; 2 times/week
/	Executive function training	Object sorting task / grocery shopping or purchasing vegetables	30 min/session; 2 times/week		
No participation in any social activities in the past month (including chatting with neighbors, playing cards, volunteering, etc.)	Psychosocial support	Participate in community activities / volunteer activities/ family visits	1 time/day		

In the tailoring factor column, a slash (/) indicates that the intervention component is delivered by default (i.e., without any additional condition) to all participants at the corresponding risk level. Other entries (e.g., SPPB≥10, frailty) indicate conditionally delivered components—they are automatically recommended or pushed to a participant only if the corresponding clinical or functional factors met, based on baseline or dynamically reassessed data. SPPB, Short Physical Performance Battery; BMI, body mass index; DASH, Dietary Approaches to Stop Hypertension; MMSE, Mini-Mental State Examination; PSQI, Pittsburgh Sleep Quality Index. *The recommended frequency refers only to the number of check-ins required per week for a fixed dietary pattern or nutrient supplementation task. However, dietary pattern and nutrient supplementation should be maintained daily.

To improve acceptability and usability of the intervention among older adults, digital materials were redesigned in age-friendly formats. To enhance compliance and ensure correct performance, a series of illustrated demonstration videos were formulated by rehabilitation professionals, along with simple verbal instructions. Cognitive and psychological content was delivered with animated videos. The use of large letters and graphical illustrations helped in better readability of nutrition and social support material.

Once the risk assessment of the disability of a participant is done, the platform implements a pre-defined stratified algorithm for the creation of an individualized intervention plan. The plan can also be changed based on the participant’s preferences. All the plans offer a set of intervention tasks across the four domains along with recommendations on how often to train, at what intensity, what to eat, safety and follow-up reminders. Throughout the intervention period, the platform will continuously collect information on user engagement, including task completion, check-in records, and follow-up responses. The data will help in monitoring the adherence, making the required alteration of the intervention plan in a timely manner, and improving the precision of subsequent recommendations.

The entire duration of the intervention will be 6 months long. During the intervention, participants will undergo periodic reassessment of disability risk related factors and risk level, and the intervention plan will be adjusted accordingly. The frequency of reassessment and plan adjustment will depend on the participant’s current risk level: every 3 months for low-risk participants, every 2 months for moderate-risk participants, and once per month for high-risk participants. If a participant’s risk level changes, the participant may move to another intervention risk tier, and the intervention packages will be modified according to the updated risk level. This dynamic adjustment will be used only to tailor the intervention during follow-up and will not alter the original randomization allocation.

#### 2.4.2 Control group

Participants of the control group will be provided with the regular community-based geriatric health management services delivered by community health centers. These services are provided according to the National Basic Public Health Service Program and the Zhejiang Provincial Basic Public Service Standards (2021 version) and therefore represent the standard care routinely available to older adults in the participating communities.

Standard care generally includes routine health education and basic lifestyle counseling provided by general practitioners and community health workers. The content may cover healthy aging, chronic disease prevention and control, smoking cessation, physical activity, nutrition, and basic dietary and exercise advice. The frequency, content, and format of standard care will follow these established routine procedures rather than being determined by the study team. However, no additional study-specific intervention, structured risk-stratified management, individualized monitoring, or extra follow-up beyond routine community practice will be arranged for the control group.

And participants in the control group will not have access to the digital platform, risk-stratified intervention protocols, individualized monitoring, or structured feedback mechanisms provided to the intervention group. For ethical reasons, participants in the control group will be offered access to the digital platform after the study period has been completed.

### 2.5 Measures

#### 2.5.1 Disability risk factors assessment

Based on the disability risk prediction model developed by the research team, a total of 29 factors will be assessed across six domains: sociodemographic characteristics, lifestyle factors, family and economic support, comorbidities and hospitalization, health status and functional limitations, and physical examinations. Sociodemographic characteristics include age, gender, education level, retirement status, and history of accidents or falls. Lifestyle factors include smoking status and participation in social activities. Family and economic support includes financial support. Comorbidities and hospitalization include history of 7 specific chronic conditions, namely hypertension, diabetes, chronic lung diseases, psychiatric illnesses, heart disease, digestive diseases, and arthritis, as well as hospitalization last year. Health status and functional limitations include self-reported physical limitations, including difficulty in extending arms, lifting weights, picking up a small coin, climbing stairs, walking for 100 meters, standing up from chair, and stooping, as well as self-reported orientation and depression. Physical examinations include Body Mass Index (BMI), waist circumference, side-by-side stand test and tandem test. Detailed assessment procedures and definitions are provided in Supplementary Table 3.

#### 2.5.2 Outcome measure

##### 2.5.2.1 Primary outcome

The primary outcome of this study is physical performance, assessed using the Short Physical Performance Battery (SPPB) (30). The SPPB is a widely used and validated instrument for assessing lower extremity function in older adults. It consists of three components: standing balance, gait speed and lower limb strength assessed using the repeated chair stand test. Gait speed was measured over a 3-meter walk. Each component is scored from 0 to 4, resulting in a total score ranging from 0 to 12, with higher scores indicating better physical performance. The assessment will be conducted by trained evaluators following standardized procedures. Participants will be asked to complete each component test under supervision, and the total SPPB score will be calculated by summing the three sub-scores. The stopwatch used was from Luotai, model XJ-894. The SPPB will be assessed at baseline (T0), 3 months after the start of the intervention (T1), and 6 months after the start of the intervention (T2).

##### 2.5.2.2 Secondary outcomes

Secondary outcomes include incidence of disability, disability risk score, and intervention adherence.

###### 2.5.2.2.1 Incidence of disability

Disability will be assessed using Basic Activities of Daily Living instruments (BADL) (26, 31) and Instrumental Activities of Daily Living (IADL) (25). Disability is defined as the onset of new or worsening dependency in performing basic or instrumental daily activities during the study period. BADL includes dressing, bathing, eating, transferring in and out of bed, toileting, and urinary and fecal continence. IADLs consisted of five items, including doing housework, cooking, shopping, taking medication, and handling finances. For each item, participants will be asked to rate their ability using four response options: no difficulty, difficulty but able to complete independently, difficulty and requiring assistance, or unable to complete. Each item will be dichotomized as 0 for no difficulty and 1 for any difficulty, need for assistance, or inability to perform the activity. Disability will be defined as difficulty in performing at least one BADL or IADL item. The incidence of disability will be determined at 6 months (T2) as the proportion of participants who develop disability among those who are non-disabled at baseline.

###### 2.5.2.2.2 Disability risk score

The disability risk score is a continuous variable ranging from 0 to 1, derived from a previously validated prediction model. A higher score indicates a greater predicted risk of developing disability. The score will be assessed at baseline (T0), 3 months (T1), and 6 months (T2).

###### 2.5.2.2.3 Intervention adherence

Adherence will be followed using records generated by the DRPP. Participants will check in on the platform after completing scheduled intervention activities, such as exercise sessions or diet-related assignments. The adherence rate will be calculated by dividing the number of activities completed by the number scheduled during the intervention period.

### 2.6 Data collection and management

Data collection will be carried out by trained study team members uniformly across all study sites who will follow special procedures. The primary sources of data will be structured self-report questionnaires, in-person performance tests, and routine records generated automatically by the digital intervention platform.

Data from self-reports will be collected online via the platform. Participants will receive the links to the questionnaires via the mobile app and are to fill the questionnaires by themselves. The trained assessor will measure SPPB and other physical performance outcomes face-to-face in a healthcare community setting. The designated research personnel will record the results of each evaluation on the platform. Data regarding engagement of intervention such as task completion, daily check-ins and other adherence indicators will be extracted from the backend of the platform.

All the study data will be accumulated in a secure electronic database protected by passwords and will be accessible only to the authorized members of the research team. Data quality will be regularly monitored through predetermined methods. For example, checks for out-of-range values, missing data, etc. In the study, participants will remain anonymous. Each subject will have a unique study code, and personal identifiers will be removed from the statistical analysis datasets.

### 2.7 Statistical analysis

The main analysis will be done on an intention-to-treat (ITT) basis including all participants as randomized, regardless of their level of adherence to the intervention. Also, a sensitivity analysis will make per-protocol (PP) analyses be done.

To characterize the study population at baseline, descriptive analyses will be performed. Continuous variables that are normally distributed will be represented using their mean and standard deviation. However, skewed variables will be summarized using medians and interquartile ranges. Quantitative factors will be presented as counts and percentages.

Statistical tests will be used to assess whether study arms are comparable at baseline. Baseline characteristics, including residence, educational level, occupation, physical activity and other sociodemographic and health-related variables, will be compared. When normality assumptions are met, independent-samples t-tests will be used for continuous variables, Mann–Whitney U tests otherwise. Depending on expected counts, chi-square tests or Fisher’s exact tests will compare categorical variables.

The SPPB score will be the main endpoint. We will fit linear mixed-effects models with fixed effects that include study group, assessment time point and their interaction to evaluate intervention effects over time. Random effects will be specified for each person to account for repeated observations on the same individual. The group-by-time interaction will be used to estimate whether changes in SPPB scores differ between the intervention and control groups over the follow-up period. This approach also permits the use of available data from participants with partially missing follow-up assessments. Given the possible ceiling effect of the SPPB among high-functioning older adults, we will examine the baseline distribution of SPPB scores and report the proportion of participants with scores close to the upper limit. Sensitivity analyses excluding participants with high baseline SPPB scores will be conducted if a substantial ceiling effect is observed.

Analyses of secondary outcomes will be selected according to the type and distribution of each variable. Continuous outcomes will be analyzed using mixed-effects models or repeated-measures methods, as appropriate. For categorical outcomes, including the occurrence of disability, between-group comparisons will be conducted using chi-square tests, and logistic regression models may be used to estimate odds ratios with corresponding 95% confidence intervals. Potential confounders or clinically relevant baseline variables may be included in multivariable models when adjustment is warranted. Urban/rural residence will be considered in the adjusted analyses where appropriate. Subgroup or sensitivity analyses by urban/rural residence will be conducted, if sample size permits, to explore whether intervention effects differ between urban and rural participants.

Missing data will be handled using multiple imputation or likelihood-based methods under the missing-at-random assumption. All analyses will be conducted using R version 4.0.3 and SPSS version 29.0 (IBM Corp., Armonk, NY, USA). Statistical tests will be two-sided, with statistical significance defined as *p* < 0.05.

## 3 Discussion

The aging of the population has led to a growing prevalence of functional disability, placing increasing pressure on public health systems worldwide (32, 33). Rather than being viewed as an unavoidable consequence of aging, disability is now increasingly regarded as a modifiable and dynamic process that can potentially be delayed or prevented through early risk identification and timely intervention (32). This study targets the early stage of disability, before obvious functional decline has occurred. Taking action before disabilities present may represent an important window for functional independence maintenance and successful aging. This prevention strategy is in line with modern active health concept (34).

One key feature of this research is the risk-stratified intervention design. Numerous community-based programs for the elderly adopt uniform interventions. This generic approach is unlikely to take into account individual differences in functional capacity, disability risk, and support needs. In this study, a disability risk prediction models is used to identify participants with different levels of risk, providing the basis for stratified and more targeted intervention delivery (25). Participants will receive personalized intervention strategies according to their predicted risk levels. Individuals at high risk will receive a greater frequency, intensity, and continuity of intervention, while those at lower risk will receive mainly health promotion and functional maintenance strategies. By matching intervention intensity to individual risk, this approach may improve the efficiency of resource allocation and enhance the precision of disability prevention, consistent with emerging evidence on the value of predictive models in geriatric care (10, 32).

The use of a digital platform is another important feature of this study, as it may improve the feasibility, scalability, and sustainability of the intervention. Digital health technologies are increasingly viewed as promising approaches to support healthy aging by improving access to care, enabling continuous monitoring, and facilitating self-management among older adults (34). Evidence from previous studies also suggests that digital interventions can improve chronic disease management, health-related knowledge, self-efficacy, and health behaviors (34–36). However, the use of digital health tools in older adults requires careful consideration of accessibility, equity, and the digital divide (22, 23). Therefore, researchers and policymakers should ensure that digital tools are equitable, unbiased, accessible, and responsive to the needs of older adults (21). In the present study, the digital platform enables real-time monitoring of adherence, automated feedback, and personalized recommendations, which may help improve participant engagement and intervention effectiveness. To minimize digital exclusion, several age-friendly design features were incorporated into the platform, including large-font interfaces, pictorial instructions, and simple instructional videos. In-person assistance was also provided by healthcare professionals at community health centers, and primary caregivers were allowed to help participants use smartphones when needed. In community settings where healthcare workforce capacity may be limited, this platform-based approach may also reduce dependence on face-to-face services and support the standardized delivery of multidomain interventions.

The primary outcome of this study is the Short Physical Performance Battery (SPPB) score. The SPPB is a well-established and validated tool for assessing lower-extremity physical performance in older adults and has been widely used to predict disability and other adverse health outcomes (37, 38). A 1-point increase in the SPPB score is generally considered clinically meaningful and has been associated with a lower risk of limitations in activities of daily living and future hospitalization (28, 29). Therefore, evaluating changes in SPPB scores before and after the intervention will allow this study to assess whether a digital, risk-stratified intervention can improve physical function and delay disability onset among older adults.

This study has several strengths. Firstly, the randomized multicenter design enhances the methodological rigor and potential generalizability of the findings. Secondly, the intervention uses a disability risk prediction model to tailor the support they receive to their predicted levels of risk. The digital platform allows for the standardization of intervention delivery, adherence monitoring, feedback automation, and fidelity monitoring. These features may improve both the precision and scalability of disability prevention in the community settings.

There are several limitations should also be noted. At first, the follow-up period of 6 months may not be insufficient to fully capture incident disability or determine the sustained effects of the intervention on disability prevention. Longer-term studies are needed to evaluate whether short-term improvements in physical performance can translate into reduced disability incidence over time. Second, moreover, while the platform contains age-friendly design elements and healthcare professionals and caregivers’ assistance, some seniors may still be affected by digital literacy and smartphone access on participation and adherence (39). Third, it is impossible to blind participants and intervention providers due to the intervention’s nature, which may increase performance bias as the study’s risk of bias. Lastly, loss-to-follow up will occur probably during the study period and that might produce bias. Statistically appropriate methods will be used to mitigate its effect if any.

## 4 Conclusion

In summary, this randomized multicenter trial aims to evaluate the efficacy of a digital platform-based, stratified intervention for the prevention of disability among community-dwelling older adults. The approach adopted in this study has the potential to offer a feasible and scalable model for early disability prevention in community settings, with the combination of risk stratification, tailored multidomain interventions and digital delivery. The results of this research will facilitate the formulation of personalized, sustainable strategies to address healthy aging and disability, an important global concern.
