# Supplementary material for: A digital platform-based stratified intervention for preventing disability risk in community-dwelling older adults: study protocol for a randomized multicenter trial
Source: Front Med (Lausanne). 2026 Jul 30;13:1890107. doi: 10.3389/fmed.2026.1890107 (PMC13467830; doi:10.3389/fmed.2026.1890107)
Supplement: Supplementary file 1 [file Data_Sheet_1.DOCX]

Supplementary Material

# SUPPLEMENTARY TABLE 1 The measures for tailoring factors of intervention

| **Factors** | **Measures** |
| --- | --- |
| Frailty | Fried frailty phenotype was used to assess frailty. It consists of five criteria: unintentional weight loss, self-reported exhaustion, weakness assessed by grip strength, slow walking speed, and low physical activity. Participants meeting three or more criteria were classified as frail. |
| SPPB | Short Physical Performance Battery consists of three components: standing balance, gait speed and lower limb strength assessed by the repeated chair stand test. Gait speed was measured over a 3-meter walk. Each component is scored from 0 to 4, resulting in a total score ranging from 0 to 12, with higher scores indicating better physical performance. |
| BMI | BMI was calculated as weight in kilograms divided by height in meters squared. |
| Balance capacity | The participant is instructed to perform side-by-side test, semi-tandem test and full-tandem test to at least 10 seconds.  Balance ability indicates the assigned highest balance level of the respondent.  Grade 1 = did not complete the semi-tandem nor side-by-side test to the maximum time.  Grade 2 = did not complete the semi-tandem test to the maximum time, but did complete the side-by-side test to the maximum time.  Grade 3 =completed the semi-tandem test to the maximum time, but did not complete the expected full-tandem test time.  Grade 4 = completed the semi-tandem test and full-tandem test to the maximum time. |
| Muscle strength | Muscle strength was assessed using manual muscle testing (MMT). Muscle strength was graded on a 0–5 scale as follows:  Grade 0 = no visible or palpable muscle contraction;  Grade 1 = slight palpable muscle contraction without joint movement;  Grade 2 = full range of motion possible only with gravity eliminated;  Grade 3 = full range of motion against gravity but not against resistance;  Grade 4 = movement against gravity and moderate resistance;  Grade 5 = movement against gravity and full resistance. |
| MMSE | Cognitive function was measured with the Mini-Mental State Examination (MMSE). It evaluates five domains of cognitive function: orientation, registration, attention and calculation, recall, and language ability. The total score ranges from 0 to 30, with higher scores indicating better cognitive function. |
| PSQI | Sleep quality was evaluated using the Pittsburgh Sleep Quality Index (PSQI). The PSQI comprises seven components: perceived sleep quality, time taken to fall asleep, sleep duration, habitual sleep efficiency, sleep-related disturbances, use of sleep medication, and daytime dysfunction. Each component is rated from 0 to 3, and the component scores are summed to generate a global score ranging from 0 to 21. Higher global scores indicate poorer sleep quality. |

# SUPPLEMENTARY TABLE 2 Intervention description

| **Intervention component** | **Description** | **Video guide** | **Picture guide** |
| --- | --- | --- | --- |
| *1.Warm-up exercise* |  |  |  |
| 1.1 Marching in place | Stand upright and march in place while swinging the arms naturally. Lift the knees comfortably and maintain an upright posture. Continue for about 10 minutes before exercise. | Yes | / |
| 1.2 Joint mobility exercises | Move the neck, shoulders, elbows, wrists, hips, knees, and ankles gently through a comfortable range of motion. Perform the movements slowly without causing pain. | Yes | / |
| 1.3 Dynamic stretching | Stretch the major muscle groups using slow and controlled movements before exercise. Avoid bouncing or overstretching. | Yes | / |
| *2.Aerobic exercise* |  |  |  |
| 2.1 Jogging | Jog at a comfortable pace after warming up. Adjust speed according to physical condition and stop if discomfort occurs. | Yes | / |
| 2.2 Stair climbing | Climb stairs steadily while holding the handrail if necessary. Avoid excessive speed and rest whenever needed. | No | Yes |
| 2.3 Aerobic exercise routine | Perform rhythmic whole-body movements at a moderate intensity while maintaining smooth breathing and coordinated body movements. | Yes | / |
| 2.4 Brisk walking | Walk at a slightly faster-than-usual pace while maintaining normal breathing. Adjust the pace according to individual tolerance. | No | Yes |
| 2.5 Swimming | Swim at a comfortable intensity according to ability. Ensure safety and avoid overexertion. | No | Yes |
| 2.6 Baduanjin | Perform the eight standardized movements slowly with coordinated breathing and controlled body movements. Focus on posture, flexibility, and balance while exercising within a comfortable range. | Yes | / |
| 2.7 Walking | Walk at a comfortable and safe pace according to physical condition. | Yes | / |
| 2.8 Household activities | Perform household activities, such as cleaning, sweeping, or mopping, to increase daily physical activity. | No | Yes |
| 2.9 Tai Chi | Perform slow and continuous movements with coordinated breathing, smooth weight shifting, and controlled body posture. Focus on balance, flexibility, and body coordination while exercising at a comfortable pace. | Yes | / |
| *3.Resistance exercise* |  |  |  |
| 3.1 Chest expansion exercise | Raise both arms to shoulder height and gently move them backward to expand the chest, then slowly return to the starting position. | Yes | / |
| 3.2 Straight arm abduction | Raise both straight arms sideways to shoulder level, pause briefly, and slowly lower them. | Yes | / |
| 3.3 Half-squat stand exercise | Sit on a stable chair, stand up slowly without using the hands if possible, and then sit down in a controlled manner. | Yes | / |
| 3.4 Heel raise | Stand behind a chair for support if needed. Slowly rise onto the toes, hold briefly, and lower the heels slowly. | Yes | / |
| 3.5 Seated leg extension | Sit upright on a chair. Slowly extend one knee until the leg is straight, hold briefly, then lower it and repeat with the other leg. | Yes | / |
| *4.Balance exercise* |  |  |  |
| 4.1 Static balance exercise | Practice standing with feet together, semi-tandem, tandem, or on one leg according to ability. Hold a stable support if needed. | Yes | / |
| 4.2 Dynamic balance exercise | Practice stepping over obstacles, walking in a figure-eight pattern, and tandem walking heel-to-toe according to ability. Hold a stable support or have supervision if needed. | Yes | / |
| *5.Dietary patterns* |  |  |  |
| 5.1 Mediterranean diet | Emphasize vegetables, fruits, whole grains, legumes, nuts, olive oil, and fish, while limiting red meat and processed foods. | No | Yes |
| 5.2 High-protein diet | Increase high-quality protein intake from fish, eggs, lean meat, dairy products, soy products, and legumes according to nutritional needs. | No | Yes |
| 5.3 DASH diet | Consume more fruits, vegetables, whole grains, and low-fat dairy products while reducing sodium, sugar, and saturated fat intake. | No | Yes |
| 5.4 High-calorie diet | Increase total energy intake with nutrient-dense foods, especially for older adults who are underweight or at risk of malnutrition. | No | Yes |
| 5.5 Low-glycemic diet | Choose low-glycemic foods such as whole grains, legumes, vegetables, and unsweetened foods, while reducing refined carbohydrates and sugary foods. | No | Yes |
| 5.6 Elder-friendly diet | Provide soft, easy-to-chew, and easy-to-digest foods while ensuring adequate energy and nutrient intake. | No | Yes |
| *6.Sleep management* |  |  |  |
| 6.1 Healthy sleep education | Maintain regular sleep and wake times, create a quiet and comfortable sleep environment, avoid caffeine and electronic device use before bedtime, and establish healthy bedtime routines. | No | Yes |
| 6.2 Relaxation and meditation training | Practice slow breathing, progressive muscle relaxation, or guided meditation in a quiet environment to reduce stress and promote relaxation before sleep. | Yes | No |
| *7.Cognitive training* |  |  |  |
| 7.1 Episodic memory training | Recall recent events or personal experiences in chronological order, focusing on details such as people, places, and activities to strengthen episodic memory. | Yes | No |
| 7.2 Working memory training | Perform simple mental tasks that require temporarily storing and manipulating information, such as remembering and repeating numbers or words. | Yes | No |
| 7.3 Associative memory training | Improve memory by linking new information with familiar people, objects, images, or daily experiences through association techniques. | Yes | No |
| 7.4 Dual-task walking | Walk at a comfortable pace while counting each step aloud or silently to improve divided attention and cognitive-motor coordination. | Yes | No |
| 7.5 Deep breathing exercise | Inhale slowly through the nose and exhale gently through the mouth while maintaining a relaxed posture. Repeat for several minutes to improve attention and relaxation. | Yes | No |
| *8.Executive function training* |  |  |  |
| 8.1 Object sorting task | Sort household items into categories based on their characteristics, such as color, size, or function, to improve planning and organization skills. | No | Yes |
| 8.2 Grocery shopping or purchasing vegetables | Select and purchase daily groceries or vegetables according to planned needs while organizing items efficiently to promote planning and decision-making skills. | No | Yes |
| *9.Psychosocial support* |  |  |  |
| 9.1 Community activities | Encourage participation in community-based activities to increase social interaction, engagement, and emotional well-being. | No | Yes |
| 9.2 Volunteer activities | Participate in appropriate volunteer activities according to individual ability to enhance social participation and sense of purpose. | No | Yes |
| 9.3 Family visits | Encourage regular visits or communication with family members to strengthen social support and reduce loneliness. | No | Yes |

**SUPPLEMENTARY TABLE 3 The detailed measures for disability risk factors**

| **Domains** | **Factors** | **Measures** |
| --- | --- | --- |
| Sociodemographic variables | Age | Years |
|  | Gender | 0=Male  1= Female |
|  | Education | 1= Less than lower secondary education  2= Upper secondary & vocational training  3= Tertiary education |
|  | Retirement status | 0= engage in agricultural work, non-agricultural employed work, non-agricultural self-employed work, or non-agricultural unpaid family business work, or unemployed or never worked per their labor force status.  1= have retired per their labor force status. |
|  | History of accidents or falls | 0= having never received medical treatment as the result of a traffic accident or another kind of major accidental injury.  1= having ever received medical treatment as the result of a traffic accident or another kind of major accidental injury. |
| Lifestyle variables | Smoking | 0= never smoke or quit smoking  1= smoke now |
|  | Social participation | 0 = did not participate in any of the social activities in the past month.  1= participated in at least one of the social activities, including: interacted with friends, played Ma-jong, played chess, played cards, or went to community club, went to a sport, social, or other kind of club, took part in a community-related organization, done voluntary or charity work or attended an educational or training course. |
| Family and economic variables | Economic support | 0= never receive financial support from their children  1=have received financial support from their children |
| Comorbidities and hospitalization | Hypertension | 0= don’t have hypertension  1= have hypertension |
|  | Diabetes | 0= never have diabetes  1= have diabetes or high blood sugar |
|  | Chronic lung disease | 0= don’t having chronic lung disease such as chronic bronchitis or emphysema (excluding tumors, or cancer)  1= having chronic lung disease such as chronic bronchitis or emphysema (excluding tumors, or cancer) |
|  | Psychiatric illnesses | 0= never have any emotional, nervous, or psychiatric problems  1= have any emotional, nervous, or psychiatric problems |
|  | Heart disease | 0= never have heart attack, coronary heart disease, angina, congestive heart failure, or other heart problems  1= have heart attack, coronary heart disease, angina, congestive heart failure, or other heart problems |
|  | Digestive disease | 0= never have any stomach or other digestive disease (except for tumor or cancer)  1= have any stomach or other digestive disease (except for tumor or cancer) |
|  | Arthritis | 0= never have arthritis or rheumatism  1= have arthritis or rheumatism |
|  | Hospitalization last year | The number of times that the respondent has received inpatient care in the past year. |
| Physical examinations | BMI | Body Mass Index (kg/m2) |
|  | Waist circumference | waist measurement in centimeters |
|  | Side-by-side stand | The respondent was instructed to stand with feet together, side-by-side, for 10 seconds. The respondent was allowed to use their arms, bend their knees, or move their body to maintain balance. Only respondents who participated in the semi-tandem test and could not maintain balance for 10 seconds in the semi-tandem test performed the side-by-side test.  0= the respondent maintained their balance below 10 seconds  1= the respondent's time more than 10 seconds (It also defaults to respondents who have completed either the semi-tandem standing test or the full-tandem test.) |
|  | Tandem stand | The respondent was instructed to stand with the heel of one foot in front of and touching the toes of the other foot for about 10 seconds.  0= the respondent maintained their balance below 10 seconds  1= the respondent's time more than 10 seconds. |
| Health status and functional limitations | Walking for 100 meters | 0= did not have any problems with walking 100m  1= have some difficulty with walking 100m or could not finish |
|  | Stooping | 0= did not have any problems with stooping kneeling, or crouching  1= have some difficulty with stooping kneeling, or crouching or could not finish |
|  | Climbing stairs | 0= did not have any problems with climbing several flights of stairs without resting  1= have some difficulty with climbing several flights of stairs without resting or could not finish |
|  | Standing up from chair | 0= did not have any problems with getting up from a chair after sitting for long periods  1= have some difficulty with getting up from a chair after sitting for long periods or could not finish |
|  | Difficulty in extending arms | 0= did not have any problems with reaching arms above shoulder level  1= have some difficulty with reaching arms above shoulder level or could not finish |
|  | Lifting weights | 0= did not have any problems with lifting or carrying weights over 5kg  1= have some difficulty with lifting or carrying weights over 5kg or could not finish |
|  | Picking up a small coin | 0= did not have any problems with picking up a coin from the table  1= have some difficulty with picking up a coin from the table or could not finish |
|  | Self-reported orientation | 0=the respondent can recall the day of the month, the month, the year and the day of the week correctly.  1=the respondent is unable to recall correctly any items, including the day of the month, the month, the year and the day of the week. |
|  | Depression | The 10-item Centre for Epidemiologic Studies Depression (CESD-10) Scale was used. The total score is calculated by finding the sum of 10 items. Any score equal to or above 10 is considered depressed.  0= total score less than 10  1= total score equals to or above 10 |
